# DNA replication roadblocks caused by Cascade interference complexes are alleviated by RecG DNA repair helicase

**DOI:** 10.1080/15476286.2018.1496773

**Published:** 2018-08-10

**Authors:** Tom Killelea, Michelle Hawkins, Jamieson L. Howard, Peter McGlynn, Edward L. Bolt

**Affiliations:** a School of Life Sciences, Queen’s Medical Centre, University of Nottingham, Nottingham, UK; b Department of Biology, University of York, York, UK

**Keywords:** CRISPR-Cas, Cascade, RecG, adaptation

## Abstract

Cascade complexes underpin *E. coli* CRISPR-Cas immunity systems by stimulating ‘adaptation’ reactions that update immunity and by initiating ‘interference’ reactions that destroy invader DNA. Recognition of invader DNA in Cascade catalysed R-loops provokes DNA capture and its subsequent integration into CRISPR loci by Cas1 and Cas2. DNA capture processes are unclear but may involve RecG helicase, which stimulates adaptation during its role responding to genome instability. We show that Cascade is a potential source of genome instability because it blocks DNA replication and that RecG helicase alleviates this by dissociating Cascade. This highlights how integrating in vitro CRISPR-Cas interference and adaptation reactions with DNA replication and repair reactions will help to determine precise mechanisms underpinning prokaryotic adaptive immunity.

## Introduction

CRISPR-Cas prokaryotic adaptive immunity protects cells from predation by phage and limits movement of mobile genetic elements (MGEs, e.g. plasmids) between cells (reviewed most recently in [1]. Immunity derives from host cell CRISPR loci that store DNA from previously encountered invader MGEs as fragments called ”spacers” that are precisely interspersed between repeat DNA sequences. Transcription of CRISPR and subsequent processing of RNA transcripts generates CRISPR RNA (crRNA) that matches originating MGE DNA sequence. Immunity is delivered when crRNA incorporated into CRISPR-Cas ‘Interference’ complexes is targeted to MGE DNA leading to its destruction by nucleases.

CRISPR-Cas immunity therefore relies on insertion of spacer DNA into CRISPR loci. This occurs by processes collectively called ‘Adaptation’ that involve the capture of MGE DNA fragments and their subsequent integration into a CRISPR locus. Adaptation in *E. coli* is dependent on Cas1 and Cas2 proteins forming an oligomer of two Cas1 dimers held together by a Cas2 dimer [2,3]. This forms a pre-integration complex with a flayed duplex DNA molecule that positions DNA 3´ OH groups appropriately for integration into CRISPR as a new spacer [4,5]. Integration occurs *via* transesterification reactions that have been elucidated in detail [6–9]. Adaptation events prior to DNA integration require DNA pre-processing into molecules suitable for capture by Cas1-Cas2. It is not clear how this occurs but genetic analysis has implicated various host cell nucleases and helicases including involvement of enzymes from host DNA replication and DNA repair pathways [10–12]. The genetic requirements for adaptation also vary according to whether Cas1-Cas2 is establishing new immunity when there is no interference because an MGE has not been previously encountered (‘naïve’ adaptation), or if Cas1-Cas2 is updating immunity after interference has recognized an MGE (‘targeted/primed’ adaptation).

‘Primed’ adaptation [13,14] and ‘targeted’ adaptation [15,16] are triggered by interference reactions catalysed in *E. coli* by ‘Cascade’ ribonucleoprotein complexes. Cascade recognizes MGE DNA by forming an R-loop of crRNA base-paired to one MGE DNA strand and the other is displaced as single-stranded DNA [17,18]. This culminates in recruitment of Cas3 nuclease that destroys MGE DNA [14,19]. *E. coli* Cascade is hetero-oligomer of five proteins assembled as Cse1-(Cse2)_2_-(Cas7)_6_-Cas5-Cas6e around a single crRNA payload that comprises a 32 nucleotide spacer sequence flanked by a few nucleotides of repeat sequence [17,20–22]. Major events of Cascade interference that lead to R-loop formation on MGE DNA begin with Cascade sampling dsDNA through electrostatic contacts between a lysine ‘vice’ of two Cas7 subunits and the phosphate backbone of the dsDNA [23]. The N-terminal domain of Cse1 recognizes a trinucleotide sequence within the target DNA called a Protospacer Adjacent Motif (PAM) and in so doing stabilises the interaction between Cascade and dsDNA [24,25] whilst destabilising the DNA duplex enabling invasion of crRNA and R-loop formation [17,26,27]. A stable R-loop can be established comprising 18–25 base pairs of RNA-DNA hybrid [28] that results in a conformational change to Cascade Cse1, facilitating recruitment of Cas3 for MGE degradation [26,29]. How these events stimulate adaptation is much less clear, although recognition of an MGE target and stable R-loop formation by Cascade are both important [28,30]. Genetic analysis in *E. coli* also implicated DNA repair enzymes RecG, PriA and DNA polymerase I in promoting adaptation when Cascade was designed to target phage λ [12]λ A model was proposed that RecG helicase promotes adaptation as a consequence of its response to genome instability when DNA replication is blocked by Cascade interference complexes. Here we show that DNA replication, Cascade interference and DNA repair can be reconstituted *in vitro* as an integrated system and that RecG interacts with Cascade R-loop complexes and removes them.

## Results and discussion

Genetic analysis has implicated RecG helicase in promoting primed and targeted adaptation by CRISPR-Cas systems in *E. coli* and *P. aeruginosa* [12,31]. We proposed that Cascade-catalysed R-loop interference reactions that target MGE DNA are a barrier to MGE DNA replication. This leads to recruitment of RecG as part of host cell responses to genome instability, and promotes adaptation. We began investigating this *in vitro* using defined DNA substrates and purified proteins.

**Figure 1.**
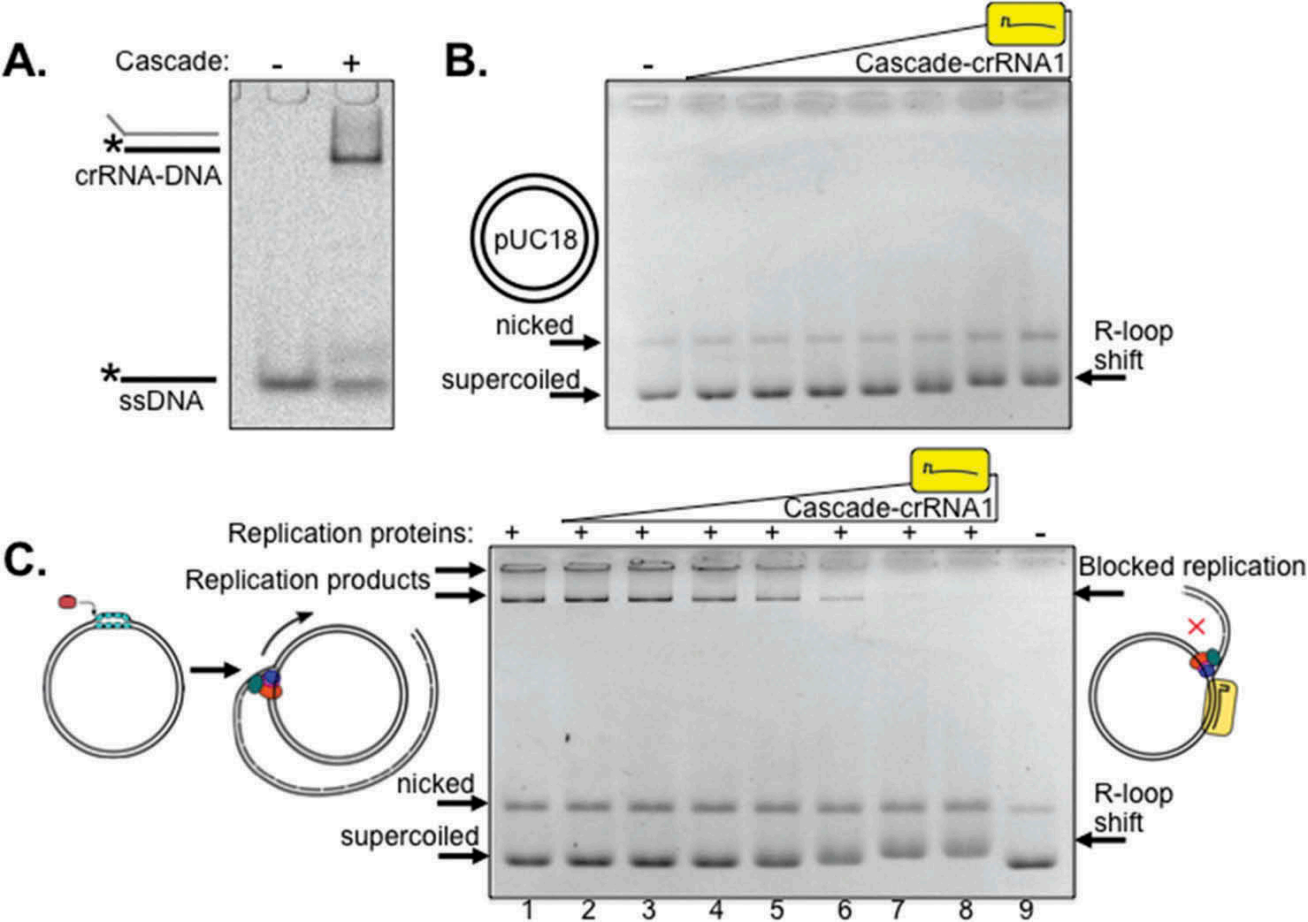
*E. coli* Cascade interference complex blocks plasmid DNA replication. (A). EMSA ‘band-shift’ of Cy5-end labelled ssDNA that is complementary to crRNA1 purified within the Cascade complex (500 nM). (B). Agarose gel (0.8%) showing R-loop plasmid mobility shifts when Cascade-crRNA1 was titrated into pUC18 that migrated as supercoiled and nicked DNA as indicated (50 ng). Cascade was used at 4, 8, 16, 32, 64, 125 and 250 nM. (C). Titration of Cascade-crRNA1 into pUC18 plasmid DNA replication reactions cause loss of replication product. Full DNA replication product is shown in (lane 1), and Cascade-crRNA1 was used at the same concentrations as in part (B).

A Cascade complex was purified containing crRNA (crRNA1) to target 32 base pairs within *lacZ* of pUC18 plasmid to determine if this had any effect on plasmid DNA replication by purified *E. coli* replisome proteins (Figure 1). Targeting of Cascade-crRNA1 to *lacZ* was confirmed by hybridisation of crRNA to cognate DNA in EMSAs (Figure 1A), and by binding to pUC18 identifiable as pronounced altered mobility of supercoiled pUC and subtly shifted mobility of nicked pUC indicating R-loop formation [32] (Figure 1B). Replication assays were performed by loading the *E. coli* replisome onto pUC18 and observed as high molecular mass DNA products within agarose gels (Figure 1C, lane 1). Plasmid replication is initiated in these assays from DnaC810 loading DnaB onto SSB-coated ssDNA generated at nicks or when DNA is supercoiled [33,34]. Replication products disappeared on addition of increasing concentrations of Cascade-crRNA1 (lanes 2–8) corresponding to altered pUC18 mobility caused by Cascade R-loop formation (Figure 1C lanes 2–8).

**Figure 2.**
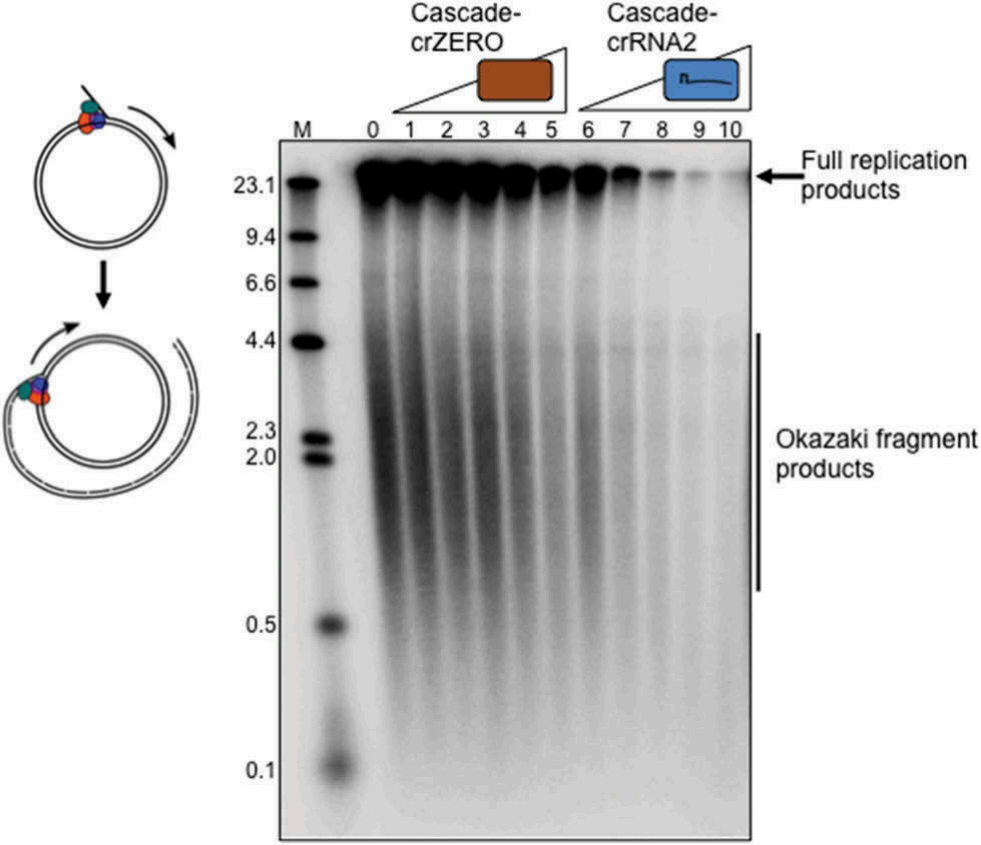
**I**nhibition of M13 DNA replication by Cascade requires a targeting crRNA. Replication reactions on nicked plasmid DNA are initiated from a flap engineered into M13 and proceed by rolling circle replication, as illustrated in the cartoon left. Full length and lagging strand (Okazaki) DNA replication products are shown in lane 0, that lacks Cascade-crRNA. Titration of Cascade-crZERO that cannot target M13 with crRNA has little effect on replication product formation but titration of Cascade-crRNA2 that does target M13 DNA (see methods) caused a substantial decrease in observable product. Cascade protein complexes were each used at 4, 8, 16, 32 and 64 nM as indicated.

Replication blockage was also observed by using an alternative interference complex (Cascade-crRNA2) that binds to a target sequence within a nicked M13 DNA substrate (Figure 2, lanes 6 – 10). A purified Cascade complex lacking cRNA (Cascade-crZERO) and therefore unable to target M13 for binding had no significant effect on DNA replication (Figure 2 lanes 1 – 5). Cascade-crZERO was stable during purification (Supplementary Figure S1A) and gave an elution peak at the same position as for Cascade-crRNA1 during gel filtration (Supplementary Figure S1B). These results indicate that a Cascade interference complex that is bound to a single target plasmid recognition sequence prevents DNA replication. Cascade R-loop formation most likely blocks DNA replication elongation at sites specific for crRNA base pairing, since Cascade-crZERO that would bind only non-specifically to DNA, if at all, did not block replication. We are currently developing assays to identify ‘pause site’ DNA replication products that would be expected to arise at or close to sites of blockage by Cascade. Such pause DNA products would be further evidence that the elongation phase of replication is being inhibited.

**Figure 3.**
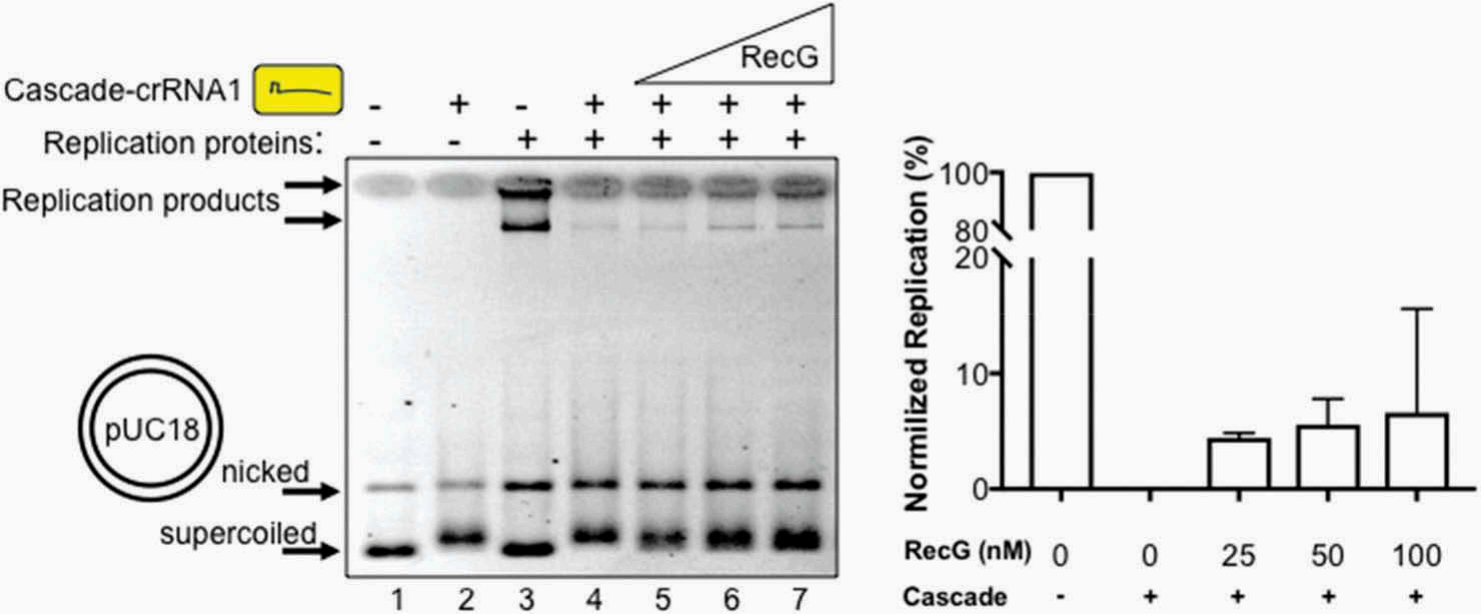
RecG alleviates replication blockage caused by a Cascade R-loop interference complex. Products of a pUC18 DNA replication assay (lane 3) were severely reduced after addition of Cascade-crRNA1 (125 nM, lane 4). Addition of RecG (25, 50, 100 nM, lanes 5–7) reproducibly stimulated replication presented as data in the graph that represents reactions in triplicate with error bars for standard deviation from the mean. The % of replication detected is expressed as a comparison with 100% replication assigned to reactions lacking Cascade-crRNA (e.g. lane 3).

Nucleoprotein complexes are a major cause of genome instability because they provoke genetic rearrangements in cells when DNA replication is blocked [35–37]. Multiple pathways have evolved to overcome replication barriers, including involvement of bacterial RecG [38,39]. The exact function of RecG in bacterial cells is not clear [40] but it can dissociate R-loop structures and also helps cell cycle progression by re-modelling replication termination barriers [38,41]. We observed that DNA replication of pUC18 (Figure 3 lane 3) that had been blocked by 125 nM Cascade-crRNA1 (lane 4) resumed with the addition of *E. coli* RecG protein. This corresponds with re-appearance of replication products (lanes 5 – 7) to about 5–10% of the total product formed when Cascade was absent from reactions, summarised in Figure 3. Resumption of replication also corresponded to mobility of pUC18 changing from slower migrating plasmid bound by Cascade (Figure 3, lanes 2, 4 and 5) to faster migrating supercoiled plasmid (lanes 6 and 7). This suggested that replication was at least partially restored by RecG displacing Cascade from pUC18.

**Figure 4.**
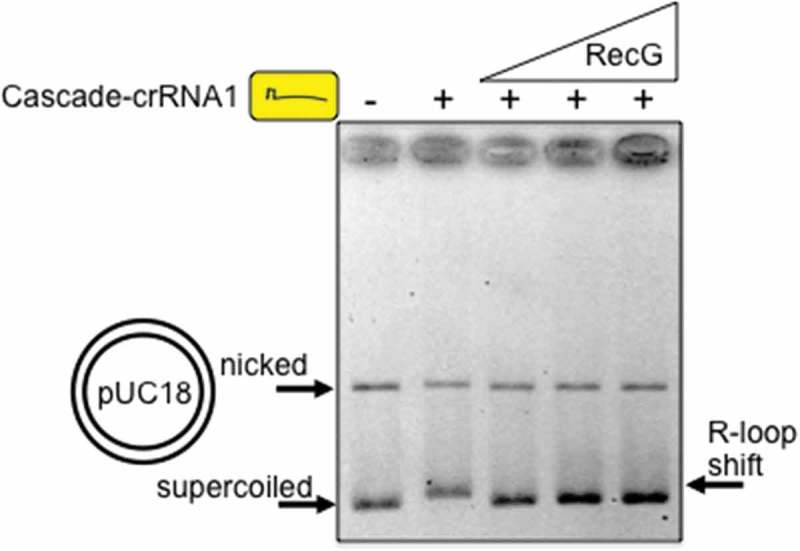
RecG (25, 50 and 100 nM) displaces Cascade R-loops from plasmid DNA. R-loops were formed between Cascade-crRNA1 and pUC18 independently of DNA replication. A ssDNA ‘trap’ in reactions prevents re-binding of Cascade-crRNA to plasmid if removed by RecG, detailed in Figure 5 and in the Methods section.

**Figure 5.**
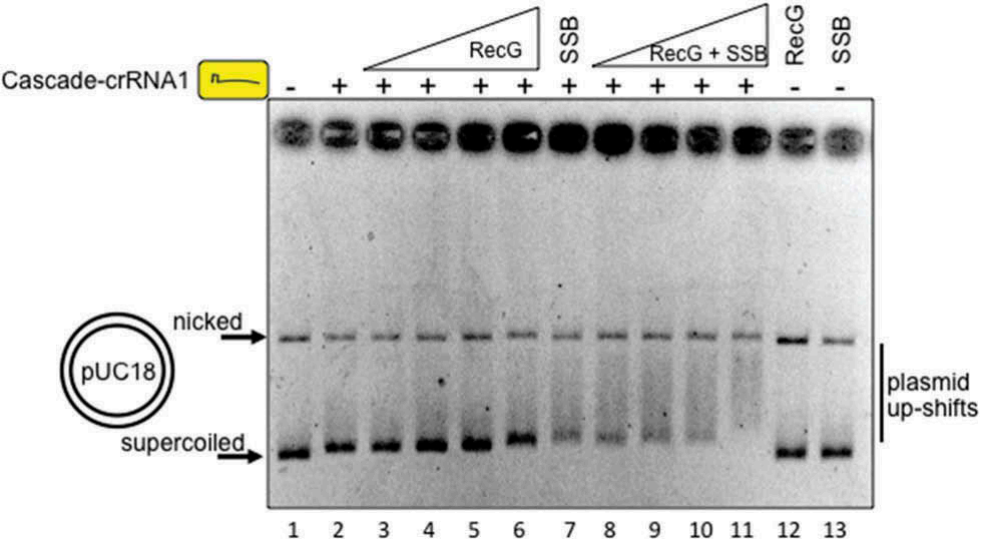
Cascade R-loops targeting pUC18 are also bound by RecG and SSB proteins. Cascade-crRNA1 and SSB concentrations were constant throughout at respectively 125 nM and 1 uM. RecG was used at 25, 50, 100 and 250 nM.

To determine if RecG alone displaced Cascade we repeated reactions in the absence of replisome proteins and again observed dissolution of Cascade R-loops on addition of RecG (Figure 4). Displacement of Cascade was observed only when a single-stranded DNA (ssDNA) ‘trap’ was used to prevent rebinding of Cascade-crRNA1 to pUC18 after its removal by RecG; the outcome of assays without a DNA ‘trap’ for Cascade are detailed further in Figure 5.

Direct interaction between *E. coli* RecG and single strand DNA binding protein SSB promotes genome stability [42,43]. This may also be relevant for recruitment of RecG to Cascade interference reactions through binding of SSB to ssDNA that is generated within R-loops. We repeated Cascade-crRNA1 reactions without the ssDNA ‘trap’ that would also be bound strongly by SSB (Figure 5). Cascade R-loops formed on pUC18 (lane 2) but their displacement by RecG was not apparent, as expected in the absence of ssDNA ‘trap’ (lanes 3 – 6). Addition of SSB alone to Cascade-pUC18 R-loop resulted in a further plasmid mobility shift (lane 7), and titration of RecG into SSB pre-bound to Cascade-pUC18 resulted in further progressively increased shifts of pUC18 into more slowly migrating plasmid DNA (lanes 8 – 11). Neither SSB nor RecG alone had any observable effect on pUC18 mobility in these gels when Cascade-crRNA1 was absent (lanes 12 and 13).

These results indicate a possible model that centres on Cascade-crRNA binding to target duplex DNA and generating a nucleoprotein R-loop. This forms a sequence-specific barrier to DNA replication that provokes genome instability and DNA repair responses. The displaced ssDNA ‘loop’ can be bound by SSB, and RecG is recruited either through its known physical interaction with SSB [42] or through RecG recognizing DNA structures within the R-loops. ATP-dependent dissociation of the cascade R-loop by RecG then generates DNA structures that are suitable for DNA capture during CRISPR-Cas adaptation, either directly by Cas1-Cas2 or mediated through another enzyme such as Cas3. This work also begins to demonstrate the feasibility of integrating *E. coli* DNA replication, CRISPR-Cas interference and DNA repair reactions by reconstitution *in vitro* to precisely determine the mechanisms involved. Further work is now required to couple these reactions to Cas1-Cas2 catalysed adaptation to determine how RecG might assist *E. coli* Cas1-Cas2 at Cascade barriers to replication *in vitro*. Similar *in vitro* analyses of naïve adaptation should be able to determine how Cas1-Cas2 can establish immunity with the aid of other DNA repair enzymes.

## Materials & methods

### Proteins

*E. coli* DNA replication proteins used were DNA polymerase III core (αϵθ), Clamp loader (τ3σσ’χψ), β clamp, SSB, DnaG and DnaB were purified as described in [36], and DnaC810 was purified according to [34]. RecG helicase protein was purified as described in [44]. Purification of *E. coli* Cascade utilized over-expression from pET-Duet (Novagen) of Cascade Cse1 subunit from multiple cloning site 1 (MCS1 cloned NcoI – EcoRI) and of Cascade subunits Cse2-Cas7-Cas5-Cas6e as an operon from multiple cloning site 2 (MCS2 cloned NdeI – XhoI). crRNA1 for assembly into Cascade was generated by synthesis of DNA based on the *E. coli* CRISPR-1 Leader-Repeat1-Spacer-Repeat2 DNA sequence (GeneArt, Life Technologies) and its cloning into pACYC-Duet. DNA was synthesised with spacer sequence 5´- AGGCCCGCACCGATCGCCCTTCCCAACAGTTG or ACCTGATTTTTGATTTATGGTCATTCTCGTTT for crRNA1 and crRNA2, respectively.. The intact Cascade complex bound to crRNA1 was recovered utilizing a Streptactin-Tag II located between Methione-1 and Alanine-2 amino acids of CasB (Cse2) in the pETDuet construct. Plasmids co-transformed into BL21 AI cells were grown at 37 °C to OD600 of 0.6 for inducing expression of Cascade and crRNA1 by addition to growth media of 0.2% L-arabinose and 0.1 mM IPTG. Growth was continued for 18 hours at 18 °C before harvesting cell pellets that were resuspended in 50 mM Tris pH 8.0 and 100 mM NaCl containing Complete^TM^ protease inhibitor cocktail (Roche). Soluble protein obtained after cell lysis by sonication and clarification by centrifugation (60 min at 17,000 rpm) was passed through a 5 ml StrepTrap column (GE Healthcare) followed by isocratic elution with 1 x buffer E (100 mM Tris pH8.0, 150 mM NaCl, 1 mM EDTA and 2.5 mM Desthiobiotin). Cascade containing fractions were further purified by HiPrep Sephacryl S-300 HR column (GE Helathcare) and eluted in storage buffer (50 mM Tris pH8.0, 150 mM NaCl, 2 mM DTT, 20% glycerol) for flash freezing and storage at −80 °C.

***In vitro* reactions: Cascade EMSAs, R-loop formation and DNA replication**

EMSA reactions binding Cascade-crRNA1 (0.5 μM) to ssDNA (20 nM) (Figure 1A) were incubated for 15 min at 25°C in 20 mM Tris pH 7.5, 75 mM NaCl, 1 mM DTT, 0.2 mM EDTA. The ssDNA used was 5´-Cy5 end-labelled of sequence complementary to crRNA1: (5´- CAACTGTTGGGAAGGGCGATCGGTGCGGGCCTCTT). Binding was analysed after electrophoresis through 5% acrylamide 1 x TBE gel for 90 minutes at 120 V and visualization on a FujiFilm FLA 3000 machine. Cascade R-loops were formed on pUC18 by incubating Cascade-crRNA1 as indicated (Figures 2 and 3) with 50 ng of plasmid in reaction buffer (40 mM Hepes pH 8.0, 10 mM DTT, 10 mM Magnesium acetate, 2 mM ATP, 0.1 mg/ml BSA) at 37 °C for 30 minutes. RecG R-loop displacement reactions were carried out as above with RecG (at indicated concentrations) and 1 μM of Cy5-ssDNA ‘trap’ or 1 μM SSB added after 15 minutes of reaction time before stopping the reactions after an additional 15 minutes by addition of 50 mM EDTA. Reactions containing the replication machinery were carried out as described above with Cascade R-loops formed on pUC18 in reaction buffer supplemented with 0.2 mM G/C/UTP and 0.04 mM dNTPs. After 15 minutes replication proteins were added: 50 nM Pol III core (αϵθ), 25 nM Clamp loader (τ3σσ’χψ), 160 nM β clamp, 1 μM SSB, 200 nM DnaG, 160 nM DnaB and 160 nM DnaC810 were added along with RecG (at indicated concentrations). In all instances reactions were quenched at T = 30 minutes by addition of 50 mM EDTA. R-loop/replication product formation was visualised following overnight migration on 0.8% agarose gels in 1 x TAE post-stained with ethidium bromide.

Nicked M13 substrate utilized in rolling circle replication assays was generated by annealing single stranded M13 with the primer oJA162 (5ʹ-TTTTTTTTTTTTTTTTTTTTTTTTTTTTTTTTTTTTGTCCACTATTAAAGAACGTGGACTCCAACG −3ʹ) and extending with exo- T4 DNA polymerase exo- (NEB) using manufacturers instructions. As this polymerase is unable to engage in strand displacement, polymerization arrested upon encountering the 5ʹ end of the primer. To ensure substrate purity excess primer, nucleotides and polymerase were separated from the DNA substrate using a Micro BioSpin-6 column (Bio-Rad). Rolling circle replication reactions were assembled on ice, 4 nM nicked M13 substrate was added to reaction buffer supplemented with 0.2 mM G/C/UTP and 0.04mM dGTP/dCTP. Replication proteins were added at concentrations described above, followed by either Cascade-crZero or Cascade-crRNA2. Reactions were pre-incubated for 2 minutes at 37°C and initiated by addition of 0.04 mM dATP/dTTP, followed 2 minutes later by 5μCi of [α^32^P]dCTP (Perkin Elmer). Reactions were quenched at T = 14 minutes by addition of ammonium acetate to a final concentration of 2.5 mM with samples immediately ethanol precipitated to remove unincorporated [α^32^P]dCTP. Pellets were resuspended in 50 mM NaOH and 30 mM EDTA prior to loading and overnight migration for 420 volt hours at 25 volts on a 0.7% denaturing agarose gel (2mM EDTA, 30 mM NaOH). The gel was fixed by washing in a solution of 5% TCA for 20 minutes followed by 10 minutes in H_2_O before drying. After overnight exposure the gel was imaged using a Personal Molecular Imager system (Bio-Rad).
