# Supplementary material for: DNA replication roadblocks caused by Cascade interference complexes are alleviated by RecG DNA repair helicase
Source: RNA Biol. 2018 Aug 10;16(4):543–8. doi: 10.1080/15476286.2018.1496773 (PMC6546356; doi:10.1080/15476286.2018.1496773)
Supplement: Supplemental Material [file krnb-16-04-1496773-s001.pptx]

## Slide 1
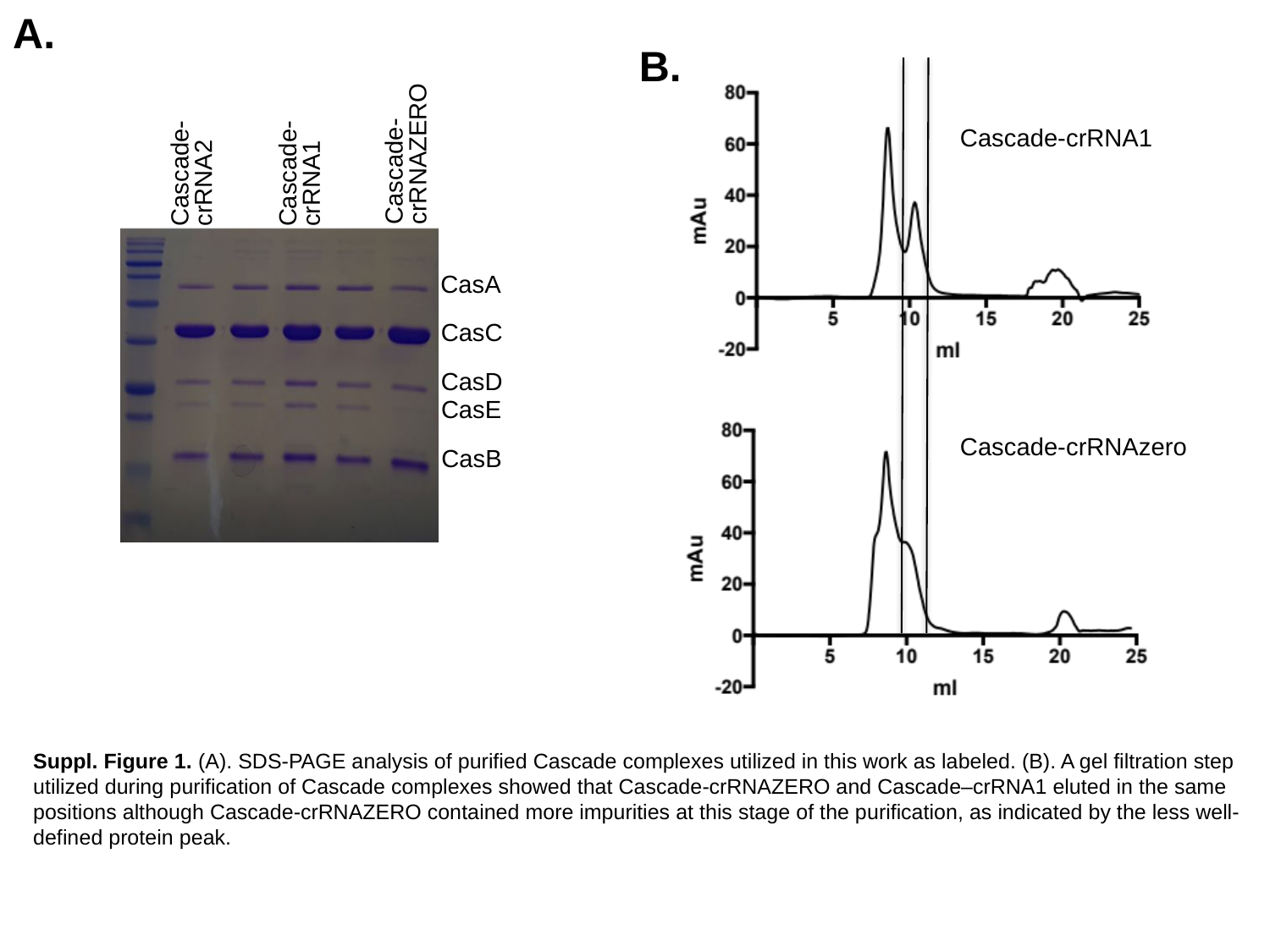

A.
B.
Cascade-crRNA1
Cascade-
crRNAZERO
Cascade-
crRNA2
Cascade-
crRNA1
CasA
CasC
CasD
CasE
Cascade-crRNAzero
CasB
Suppl. Figure 1. (A). SDS-PAGE analysis of purified Cascade complexes utilized in this work as labeled. (B). A gel filtration step
utilized during purification of Cascade complexes showed that Cascade-crRNAZERO and Cascade–crRNA1 eluted in the same
positions although Cascade-crRNAZERO contained more impurities at this stage of the purification, as indicated by the less well-
defined protein peak.
